# A Potential Adjuvant Agent of Chemotherapy: Sepia Ink Polysaccharides

**DOI:** 10.3390/md16040106

**Published:** 2018-03-28

**Authors:** Fangping Li, Ping Luo, Huazhong Liu

**Affiliations:** College of Chemistry & Environment, Guangdong Ocean University, Zhanjiang 524088, China; 15709482571@163.com (F.L.); luopingna@163.com (P.L.)

**Keywords:** Sepia ink polysaccharides, chemoprevention, antitumour, chemosensitization, anticoagulation

## Abstract

Sepia ink polysaccharide (SIP) isolated from squid and cuttlefish ink is a kind of acid mucopolysaccharide that has been identified in three types of primary structures from squid (*Illex argentinus* and *Ommastrephes bartrami*), cuttlefish *Sepiella maindroni,* and cuttlefish *Sepia esculenta* ink. Although SIP has been proved to be multifaceted, most of the reported evidence has illuminated its chemopreventive and antineoplastic activities. As a natural product playing a role in cancer treatment, SIP may be used as chemotherapeutic ancillary agent or functional food. Based on the current findings on SIP, we have summarized four topics in this review, including: chemopreventive, antineoplastic, chemosensitive, and procoagulant and anticoagulant activities, which are correlative closely with the actions of anticancer agents on cancer patients, such as anticancer, toxicity and thrombogenesis, with the latter two actions being common causes of death in cancer cases exposed to chemotherapeutic agents.

## 1. Introduction

Sepia ink, a black suspension of melanin granules, is a traditional Chinese medicine listed in the *Compendium of Materia Medica* compiled by Shizhen Li, a famous doctor at the time of the Ming Dynasty, and has been used in Asia for millennia [1]. The ancient medicine book records the treatment efficacies on heart pain and haemostasis, especially gynaecological haemostasis [1]. Based on the plentiful findings in the latest two decades, the dark ink has been proved useful and to be a kind of multifunctional bioactive marine substance as antioxidant [1,2,3,4,5,6,7,8], anti-inflammatory [9,10], anti-ulcerogenic [10,11], anti-retroviral [12], anti-hypertensive [13], antimicrobial [14,15,16,17], and anti-radiation reagent [18], and to have anticancer properties [9,17,19,20,21], as well as haematopoietic [1,18], immunoregulatory [1,4,7], procoagulant [22] and chemoprophylactic activities [1,5,6,7,8]. Sepia ink is a mixture secreted from two glands: the ink gland in the ink sac, and a mucus-producing gland that is a poorly understood funnel organ [23]. The ink contains melanin, proteins, peptidoglycans, amino acids, lipid, metals, tetrodotoxin, etc. [1,23]. The peptidoglycans are composed of sepia ink polysaccharide (SIP) and oligopeptide (SIO) [23]. SIPs derived from ink of different cuttlefishes and squids have distinct primary structures. To date, only one kind of SIO has been characterized, and this is a tripeptide consisting of glutamine (Gln), proline (Pro) and lysine (Lys), the peptide chain is *N*-Gln-Pro-Lys-*C* derived from *Sepia esculenta* ink [21].

The biological functions of melanin, proteins, amino acids, SIO, and metals in the ink have been investigated by various researchers [23]. According to the published work, in contrast, SIP has undoubtedly attracted more attention. The polysaccharide is a glycosaminoglycan that can be absorbed rapidly by the host gastrointestinal tract, and its content in serum can reach a peak at 1 h after gavage [24]. Reports have outlined the activities of this marine polysaccharide. In this paper, the biological properties based on chemopreventive, antineoplastic and chemosensitive effects, the molecular mechanisms involved, and the molecular characteristics of SIPs have been summarized.

## 2. Molecular Characteristics of SIPs

As shown in Table 1, the polysaccharide from the ink of the squid *Illex argentinus* was the first known SIP that was reported by Takaya et al. in 1994 [25]. The fucose-rich glycosaminoglycan is composed by equimolar ratios of glucuronic acid (GlcA), N-acetylgalactosamine (GalNAc) and fucose (Fuc). Its primary structure was initially determined to have a linear repeating structure of (-6GalNAcα1-3GlcAβ1-3Fucα1-)*_n_* [25], but this was amended to (-3GlcAβ1-4(GalNAcα1-3)Fucα1-)*_n_* by the discoverers themselves in their next work [26]. The main chain of the polysaccharide was a repeating unit of di-saccharide, GlcA-Fuc, branched at Fuc H-3 by GalNAc [26]. Interestingly, the SIP’s primary structure is identical to another SIP derived from squid *Ommastrephes bartrami* ink that was reported by Chen et al. [27].

Apart from the two kinds of SIPs from squids, SIPs from cuttlefish have been reported in recent years. A heteropolysaccharide was isolated from cuttlefish *Sepiella maindroni* ink using enzymolysis, anion-exchange, and gel-permeation chromatography [28]. This SIP contained GlcA, mannose (Man), GalNAc, and Fuc in a molar ratio of 1:1:2:2. Its primary structure was determined to comprise a main chain composed of a repeating unit of (-Fuc-Fuc-GalNAc-Man-GalNAc-)*_n_* and a branch of GlcA at Man H-3; the structural characteristic was (-4Fucβ1-4Fucβ1-4GalNAcα1-6(GlcAα1-3)Manα1-4GalNAcα1-)*_n_*, which differentiates it from squid ink polysaccharides.

Recently, a novel SIP was isolated from the ink of cuttlefish *Sepia esculenta* in our laboratory [29]. This polysaccharide has a unique primary structure mainly composed of galactosamine (GalN) and arabinose (Ara) in an approximate molar ratio of 1:1. The two monosaccharides account for 81.72% of the total monosaccharide mass. This SIP also contains small amounts of Fuc (9.00%), xylose (Xyl, 4.32%), Man (0.09%), glucosamine (GlcN, 1.35%), GlcA (1.98%), and galacturonic acid (GalA, 1.53%). The detailed molecular structure of this SIP will be revealed in a future report.

To date, a great number of bioactive polysaccharides have been characterized from marine organisms, including marine animals, plants, and microorganisms. Most of these reports are focused on polysaccharides from marine animals and plants. The published marine plant origin carbohydrates mainly include marine algae saccharides, such as alginates, carrageenans, and fucoidans. Chitosans, hyaluronans and chondroitin sulphates are the main polysaccharides of marine animals, and have been studied for several decades. Table 2 indicates that these polysaccharides, which have unique molecular characteristics, possess biological activities and action mechanisms [30,31,32]. Obviously, SIP is a different polysaccharide compared with other marine origin polysaccharides. Based on structure and function observations, SIP possesses specific activities that differ from the well-studied marine polysaccharides. Therefore, the following section reviews research progress of SIP properties in recent years.

## 3. Biological Activities of SIPs

SIP has been confirmed to have chemoprevention, antitumour, chemosensitization and anticoagulation activities. This section summarizes the properties listed in Table 3.

### 3.1. Chemoprevention

#### 3.1.1. Protection of the Reproductive System

With increasing incidence and mortality rates, cancer is the leading cause of death in China and is a major public health problem. Because of its large population, China’s cancer cases constitute almost 22% of global new cancer cases and close to 27% of global cancer deaths [59]. Furthermore, cancers are becoming more likely to be found in younger patients, resulting in increasing numbers of cancer patients of childbearing age. Since chemotherapy is still a major therapeutic method for cancer, anticancer agents exerting toxic effects on the reproductive system in patients of childbearing age is almost inevitable, and can potentially lead to damage and consequent infertility. Therefore, screening substances with chemopreventive properties in order to attenuate the negative effects of chemotherapeutic drugs is urgent for the treatment of the increasing number of cancer cases of childbearing age.

SIP has been verified to have chemoprophylactic actions on the reproductive system [29,48,49,50,51,52,53]. When male mice exposed to cyclophosphamide were administered SIP, the abnormal rates of their sperm declined, and the foetal abnormalities in female mice mated with them also declined, with total foetal count and average foetal count increasing [48].

The toxicity mechanisms of cyclophosphamide are complicated. Drug-induced oxidative stress and DNA strand breakage are two critical causes [60,61,62,63,64]. Cyclophosphamide-exposed mice/rats showed disruption of testicular antioxidant capacity and histopathologic changes through suppression of the nuclear factor erythroid 2 related factor 2 (Nrf2)/antioxidant response element (ARE) signalling pathway [48,49,50,51,52,60,61,62]; however, testicular functional disorders of the chemotherapeutic model animals were prevented by SIP via activation of the antioxidant signalling pathway [48,49,50,51,52]. In addition, SIP can prevent animals from cyclophosphamide-mediated mutation in vivo and H_2_O_2_/UV-induced DNA strand break in vitro [28,65]. The testicular cells of cyclophosphamide-exposed mice, including spermatogonia, Sertoli cells, and Leydig cells, were protected by SIP via repression of cyclophosphamide-induced autophagy-associated cell death and apoptosis; the mechanisms involved p38 mitogen-activated protein kinase and phosphoinositide 3-kinase (PI3K)/Akt signalling pathways [51,52,53]. Similarly, for cyclophosphamide-mediated ovarian failure, SIP also successfully inhibited follicle deletion and granule cell disruption by repressing cyclophosphamide-induced autophagy-associated cell death and apoptosis via regulation of p38 mitogen-activated protein kinase and PI3K/Akt pathways, resulting in functional rescue of the ovaries of cyclophosphamide-exposed mice [29]. These data show that SIP can prevent mice from reproductive system damage caused by cyclophosphamide-associated toxicities.

#### 3.1.2. Protection of Intestinal Tract

Cyclophosphamide-mediated augmentation of intestinal pathogenic bacterial counts and intestinal permeability was found to have a negative effect on cancer patients. Intestinal imbalance and consequent infections were consequences of the immune system disruption resulting from chemotherapy [66], and can be partly attributed to a decrease in immunoglobulin A (IgA) production due to the anticancer agent-induced reduction of IgA-producing cells [67]. Tang et al. found that, in cyclophosphamide-exposed mice, SIP could recover the balance of intestinal microflora by blocking the anticancer agent-mediated reduction of the quantity of probiotic Bifidobacterium [34]. Intestinal microbiota promote development and regulation of the acquired mucosal immune system [68,69]. As an important element of the intestinal mucosal immune system, under exposure to a chemotherapeutic agent, IgA-producing cell reduction is responsible for the imbalance in intestinal microflora. In one study, SIP promoted the expression of IL-6, IL-10, and TNF-α and up-regulated the expression of IgA J chain gene in IgA-producing cells and pIgR gene in epithelial cells. Meanwhile, SIP increased the expression of unfolded protein response effecters XBP-1s and Bip to accelerate IgA secretion. As a result, the IgA content in the intestinal tract of mice exposed to SIP was elevated [35]. A further investigation with high-throughput sequencing analysis revealed that SIPs altered the imbalance of the gut microbial ecology caused by cyclophosphamide; amounts of *Ruminococcus*, *Bilophila*, *Oscillospira*, *Dorea* and, especially, *Mucispirillum* were reduced, resulting in the repression of early disruption of the colonic surface mucus layer and an increase in the risk of inflammatory disorders [36].

The intestinal epithelium is a vital barrier contributing to preventing infection and to innate immunity, and maintaining its integrity is necessary for normal intestinal function and a healthy body. However, maintaining the integrity of the intestinal mucosa is almost impossible under exposure to chemotherapeutic drugs. Chemotherapy-induced disruption of intestinal barrier function has been confirmed [68,69]. The goblet cells, Paneth cells, and epithelial junctions (tight junctions and adherent junctions) are responsible for the integrity of the barrier, but these three important elements can be destroyed by the chemotherapeutic drug cyclophosphamide [37,38,39,40]. As a type of major epithelial cell in the small intestine, goblet cells provide first-line protection for the host against possible pathogens, which is an important part of the innate mucosal immune system. SIPs are capable of increasing quantities of goblet cells in mice to express more mucins, such as Cyto18, avoiding pathogens penetrating or colonising in the intestinal mucosa, and rescuing mucosal immunity of cyclophosphamide-treated mice [37]. Additionally, Paneth cells, another intestinal epithelial cell contributing to innate immunity by secreting antimicrobial proteins onto the mucosal surface, can be promoted by SIP to express antimicrobial proteins, including lysozyme, angiogenin-4, defensin alpha 5, and type-2 secretory phospholipase. The mechanisms depend on the relatively highly developed endoplasmic reticulum structure, not on increases in the quantity of endoplasmic reticulum, which is associated with the SIP-activated, IRE-1 mediated, XBP-1s pathway [38]. In addition, chemotherapy damages epithelial junctions and destroys the intestinal barrier, resulting in disruption of the innate immune system and consequent infections, and chemotherapeutic mucositis. Zuo et al. discovered that SIP effectively improves expression of occludin, zonulae occluden 1/2/3, claudin, cingulin, and E-cadherin genes, stabilizing tight junctions and adherent junctions, which was helpful for protecting immune function of intestinal mucosa in mice exposed to chemotherapeutic drugs [39].

Histopathological observation showed that mice treated with SIP have longer small intestinal villi, deeper crypts, and a larger ratio of villus height/crypt depth compared with cyclophosphamide-treated mice [40]. Moreover, SIP-treated mice have stronger antioxidant capacity in the intestinal tissue when compared with model mice [40].

#### 3.1.3. Protection of Other Tissues/Organs

Apart from the reproductive system and intestinal tract, chemoprevention of some other organs/tissues by SIP was also investigated in our laboratory, including liver, kidney, heart, spleen, lung, and bone marrow. SIP repressed cyclophosphamide-induced alterations of biochemistry indicators in the serum and tissues/organs of model animals, such as relative masses of liver and spleen, activities of glutamic-pyruvic transamine, glutamic-oxalacetic transaminease, catalase, lactic dehydrogenase, and creatine kinase in serum, antioxidant capacity of liver and heart, serum urea nitrogen content, peripheral blood profile including quantities of erythrocytes, leukocytes, and platelets, and haemoglobin content, as well as the DNA content in bone marrow [54,55,56].

### 3.2. Antitumour Activities

Initially, SIP’s antitumour activity was investigated by Takaya et al. in 1994 [33]. Their results showed that SIP had no inhibitory activity on Meth-A fibrosarcoma cells transplanted into BALB/c mice, but peptidoglycan had. Therefore, the researchers deduced that the antitumour activity was attributable to the complex of SIP and other components (for example, peptide or melanin) [33]. However, in the last decade, many reports have verified the antitumour effects of SIP on several types of tumours.

Unmodified naturally occurring SIPs were found to be able to inhibit both melanoma cell B16F10 [57] and human adenocarcinoma cell line MDA-MB-231 [58]. Growth and proliferation of B16F10 were repressed, tyrosinase activity and melanin production were also effectively reduced. Similarly, proliferation and migration of MDA-MB-231 were significantly blocked [58]. It is well known that polysaccharide sulphates have stronger antitumour properties. Now, several studies have also reported anticancer activities of sulphated SIP (S-SIP).

A S-SIP isolated by Chen et al. inhibited invasion and migration, but not proliferation, of human hepatocellular liver carcinoma cell line HepG2, and inhibited angiogenesis in a chick embryo chorioallantoic membrane model [41].

Another S-SIP decreased MMP-2 expression of SKOV-3 and human umbilical vein vascular endothelial cells ECV304, leading to inhibition of SKOV-3 cell penetration and ECV304 cell migration [43]. The SIP inhibits proliferation, migration, invasion, and MMP-2 expression of human epidermoid carcinoma cell line KB by inhibiting the EGFR/Akt/p38 MAPK/MMP-2 signalling pathway [44]. Furthermore, the sulphated SIP combines with the cell membrane of human ovarian cancer cell line SKOV-3, human colorectal adenocarcinoma cell line HT-29, and mouse fibroblast cell line L929 cells. In SKOV-3 cells, SIP binds to epidermal growth factor receptor (EGFR) and inhibits EGF-induced expression and activation of EGFR as well as cell migration. Consequently, the SIP suppressed EGFR-mediated p38/MAPK and PI3K/Akt/mTOR signalling pathways to inhibit migration, invasion, and MMP-2 expression of SKOV-3 cells [45].

Additionally, in vivo data showed that the derivative SIP repressed tumour growth and enhanced immune function in S180-bearing mice, also induced SKOV-3 cells apoptosis in vivo and in vitro [46]. Further investigation indicated that sulphated SIP decreased melanoma cell B16F10 pulmonary metastasis in mice models, and down-regulated expression of the intercellular adhesion molecule 1 (ICAM-1) and basic fibroblast growth factor (bFGF) in lung metastasis nodules. Furthermore, neovascularisation was suppressed in chick chorioallantoic membrane exposed to S-SIP [46]. In vitro experiments exhibited an expression reduction of ICAM-1 and bFGF in SKOV-3 and EA.hy926 cells, respectively [46]. These results suggested that S-SIP down-regulated the expression of ICAM-1 and bFGF to inhibit tumour adhesion and angiogenesis. Consequently, invasion and migration of tumour cells were restrained.

### 3.3. Chemosensitization

Combination treatment is frequently used in cancer treatment to reduce drug resistance, alleviate adverse effects, and enhance anticancer efficacy.

Currently, only two papers have reported chemosensitization by SIP. Zong et al. found that sulphated SIP increased the killing effects of cyclophosphamide on tumours and reduced the toxicity of the chemotherapy drug on the thymus in S180-bearing mice [47]. Our previous work indicated that SIP enhanced inhibition of proliferation and migration of MDA-MB-231 cells by cisplatin [58].

### 3.4. Anticoagulant and Procoagulant Activities

It has now been confirmed that chemotherapy induces hypercoagulability of blood, and consequent thrombus formation is a critical cause of cancer death, so a potential anticancer agent should possess anticoagulation properties.

Ancient Chinese medicine used sepia ink as a coagulant drug for internal haemorrhage, especially as a coagulant for gynaecology. Modern medicine, directly or indirectly, has noted the coagulant property of the ink [22,70]. Although there is no direct evidence, to date, indicating the coagulant activity of SIP, a report of the haemostatic effects of a SIP-chitosan hybrid haemostatic sponge implies that natural SIP might possess procoagulant activity [71]; however, the confusion should be removed as early as possible by future investigation.

In contrast, a paper showed anticoagulant activity of SIP. Chen et al. prepared a derivative SIP that was sulphated chemically in pyridine-sulphur-trioxide complex in a dimethyl sulphoxide system or triethylamine pyridine-sulphur-trioxide complex in a dimethyl sulphoxide system. The sulphation mainly occurred at the 4,6-positions of GalNAc, the active primary structure of the sulphated SIP was identified to be (-GlcAβ1-4(4,6-SO_4_-GalNAcα1-3)Fucα1-)*_n_*. The sulphated SIP in vitro increased partial thromboplastin time and prothrombin time, suggesting that the derivative SIP could play an anticoagulation role by inhibiting endogenous and exogenous blood coagulation processes. The sulphated SIP effectively suppressed activities of clotting factors, FIIa and FXa, mediated by antithrombin III or heparin cofactor II [42].

Other reports have shown that the sulphated group in polysaccharides has an important function with regard to anticoagulant activity; sulphation can promote anticoagulation of a non-sulphated group polysaccharide, and the degree of sulphation is positively correlated to anticoagulation [42,72]. Recently reported natural SIPs include acid mucopolysaccharides with or without a small quantity of sulphated groups. The low sulphated group content of polysaccharides has been deduced to have procoagulant activity, but an experiment has shown that sulphation-modified SIP exhibited anticoagulant activity [42], which implies that sulphated SIP is more suitable for developing an ancillary antitumour drug for cancer treatment.

## 4. Conclusions

This review summarized chemopreventive, antineoplastic, chemosensitive, and procoagulant/anticoagulant properties of SIPs, as well as their molecular characteristics. Various SIPs with distinct primary structures from different sepia inks share similar biological actions. The number of sulphated groups is crucial to the coagulant actions of SIP, with low sulphated group content leading to procoagulation activities and high sulphated group content leading to anticoagulation activities. The sulphated SIP may be an important bioactive marine substance, which could be developed as a clinical antitumour agent or chemotherapy-supplementary functional food for application in the clinical treatment of cancer.

## Figures and Tables

**Table 1:** Molecular characteristics of SIPs.

Species	Monosaccharides (Molar Ratio)	Primary Structure	Sulphate (Molar Ratio: Sulphate/Monsaccharides)	Literature
*Illex argentinus*	GlcA, GalNAc, Fuc (1:1:1)	(-3GlcAβ1-4(GalNAcα1-3)Fucα1-)*_n_*	1/3	[25,26]
*Ommastrephes bartrami*			no	[27]
*Sepiella maindroni*	Fuc, GalNAc, GlcA, Man (2:2:1:1)	(-4Fucβ1-4Fucβ1-4GalNAcα1-6 (GlcAα1-3)Manα1-4GalNAcα1-)*_n_*	unknown	[28]
*Sepia esculenta*	GalN, Ara, Fuc (5:5:1)	unknown	unknown	[29]

**Table 2:** Molecular characteristics and properties of some marine polysaccharides [30,31,32].

Species	Polysaccharides	Monosaccharides	Properties
Marine plants	alginate	l-guluronate, d-mannuronate	antibacterial, tissue regeneration
	carrageenan	d-galactose, d/l-galactose	anticoagulant, antitumour, immunomodulatory, antihyperlipidemic, antioxidant, antibacterial, antifungal, antiviral
	fucoidan	l-fucose	antitumour, anticoagulant, anti-adhensive, antiviral
Marine animals	chitosan	d-glucosamine	antimicrobial, antitumour, anti-inflammatory
	chondroitin sulphate	glucuronic, N-acetyl-galactosamine	Improving function and elasticity of the articular cartilage, hemostasis and anti-inflammation, regulation of cell development, cell adhesion, cell proliferation, cell differentiation, anticoagulation
	hyaluronan	N-acetyl-d-glucosamine, d-glucuronic acid	tissue regeneration, cell prolifernation, cell differentiation, cell migration

**Table 3:** Biological activities of SIPs.

Species	Sulfation	Properties	Targets	Literature
*Illex argentinus*	no	no antitumouractivity	Meth-A	[33]
*Ommastrephes bartrami*	no	chemoprevention	intestinal tract (mice)	[34,35,36,37,38,39,40]
	yes	antitumour	HepG2	[41]
		anticoagulant	blood (in vitro experiment)	[42]
*Sepiella maindroni*	yes	antitumour	SKOV-3, KB, HT-29, S180, B16F10	[43,44,45,46,47]
*Sepia esculenta*	no	chemoprevention	testis, ovary, spleen, kidney, liver, lung, heart, bone marrow (mice)	[29,48,49,50,51,52,53,54,55,56]
		antitumour	B16F10, MDA-MB-231	[57,58]

## References

[1] Zhong J.P., Wang G., Shang J.H., Pan J.Q., Li K., Huang Y., Liu H.Z. (2009). Protective effects of squid ink extract towards hemopoietic injuries induced by cyclophosphamide. Mar. Drugs.

[2] Saleh H., Soliman A.M., Mohamed A.S., Marie M.A.S. (2015). Antioxidant effect of *Sepia* ink extract on extrahepatic cholestasis induced by bile duct ligation in rats. Biomed. Environ. Sci..

[3] Vate N.K., Benjakul S., Agustini T.W. (2015). Application of melanin-free ink as a new antioxidative gel enhancer in sardine surimi gel. J. Sci. Food Agric..

[4] Liu H.Z., Luo P., Chen S.H., Shang J.H. (2011). Effects of squid ink on growth performance, antioxidant functions and immunity in growing broiler chickens. Asian Austral. J. Anim. Sci..

[5] Liu H.Z., Wang G., Guo Y.Z., Pan J.Q., Huang Y., Zhong J.P., Li K. (2009). *Sepia* ink extract attenuates renal injury caused by cyclophosphamide-induced oxidative stress. Chin. J. Nephrol..

[6] Wang G., Guo Y.Z., Guan S.B., Zhong J.P., Pan J.Q., Huang Y., Liu H.Z. (2009). Protective effects of sepia ink extract on cyclophosphamide-induced pulmonary fibrosis in mice. Chin. J. Mar. Drugs.

[7] Wang G., Pan J.Q., Zhong J.P., Li K., Huang Y., Wu J.L., Liu H.Z. (2009). Protective effects of sepia ink extract against cyclophosphamide-induced oxidative damage in mice spleen. Food Sci..

[8] Wang G., Liu H.Z., Wu J.L., Cao Q.W., Chen Y.P., Yang C.L., Zhong J.P. (2010). Study of sepia ink extract on protection from oxidative damage of cardiac muscle and brain tissue in mice. Chin. J. Mod. Appl. Pharm..

[9] Fahmy S.R., Soliman A.M. (2013). In vitro antioxidant, analgesic and cytotoxic activities of *Sepia officinalis* ink and *Coelatura aegyptiaca* extracts. Afr. J. Pharm. Pharmacol..

[10] Mimura T., Itoh S., Tsujikawa K., Nakajima H., Satake M., Kohama Y., Okabe M. (1987). Studies on biological activities of melanin from marine animals. V. Anti-inflammatory activity of low-molecular-weight melanoprotein from squid (Fr. SM II). Chem. Pharm. Bull..

[11] Mimura T., Maeda K., Terada T., Oda Y., Morishita K., Aonuma S. (1985). Studies on biological activities of melanin from marine animals. III. Inhibitory effect of SM II (low molecular weight melanoprotein from squid) on phenylbutazone-induced ulceration in gastric mucosa in rats, and its mechanism of action. Chem. Pharm. Bull..

[12] Rajaganapathi J., Thyagarajan S.P., Patterson Edward J.K. (2000). Study on cephalopod’s ink for anti-retroviral activity. Indian J. Exp. Biol..

[13] Kim S.Y., Kim S.H., Song K.B. (2003). Characterization of a partial purification and angiotensin-converting enzyme inhibitor from squid ink. Agric. Chem. Biotechnol..

[14] Fahmy S.R., Soliman A., Ali E.M. (2014). Antifungal and antihepatotoxic effects of sepia ink extract against oxidative stress as a risk factor of invasive pulmonary aspergillosis in neutropenic mice. Afr. J. Tradit. Complement. Altern. Med..

[15] Petkovic M.V. (2013). Determination of the antimicrobial activity of purified melanin from the ink Octopus mimus Gould, 1852 (Cephalopoda: Octopodidae). Lat. Am. J. Aquat. Res..

[16] Shi L.S., Liu H.Z., Zhong J.P., Pan J.Q. (2015). Fresh-keeping effects of melanin-free extract from squid ink on yellowfin sea bream (*Sparus latus*) during cold storage. J. Aquat. Food Prod. Technol..

[17] Kumar P., Kannan M., ArunPrasanna V., Vaseeharan B., Vijayakumar S. (2018). Proteomics analysis of crude squid ink isolated from *Sepia esculenta* for their antimicrobial, antibiofilm and cytotoxic properties. Microb. Pathog..

[18] Lei M., Wang J.F., Wang Y.M., Pang L., Wang Y., Xu W., Xue C.H. (2007). Study of the radio-protective effect of cuttlefish ink on hemopoietic injury. Asia Pac. J. Clin. Nutr..

[19] Sasaki J., Ishita K., Takaya Y., Uchisawa H., Matsue H. (1997). Anti-tumor activity of squid ink. J. Nutr. Sci. Vitaminol..

[20] Ding G.F., Huang F.F., Yang Z.S., Yu D., Yang Y.F. (2011). Anticancer activity of an oligopeptide isolated from hydrolysates of sepia ink. Chin. J. Nat. Med..

[21] Huang F., Yang Z., Yu D., Wang J., Li R., Ding G. (2012). Sepia ink oligopeptide induces apoptosis in prostate cancer cell lines via caspase-3 activation and elevation of Bax/Bcl-2 ratio. Mar. Drugs.

[22] Xie G.L., Lv C.L., Hong M.B. (1994). Experimental study of the effective mechanisms of sepia ink in promoting coagulation of blood. J. China Med. Univ..

[23] Derby C.D. (2014). Cephalopod ink: Production, chemistry, functions and applications. Mar. Drugs.

[24] Zuo T., Zhang N., Zhang Q., Shi H., Lu S., Xue C., Tang Q.J. (2016). Transportation of squid ink polysaccharide SIP through intestinal epithelial cells and its utilization in the gastrointestinal tract. J. Funct. Foods.

[25] Takaya Y., Uchisawa H., Hanamatsu K., Narumi F., Okuzaki B., Matsue H. (1994). Novel fucose-rich glycosaminoglycans from squid ink bearing repeating unit of trisaccharide structure (-6GalNAcα1-3GlcAβ1-3Fucα1-)_n_. Biochem. Biophy. Res. Commun..

[26] Takaya Y., Uchisawa H., Narumi F., Matsue H. (1996). Illexins A, B and C from squid ink should have a branched structure. Biochem. Biophy. Res. Commun..

[27] Chen S.G., Xu J., Xue C.H., Dong P., Sheng W.J., Yu G.L., Chai W.G. (2008). Sequence determination of a non-sulfated glycosaminoglycan-like polysaccharide from melanin-free ink of the squid *Ommastrephes bartrami* by negative-ion electrospray tandem mass spectrometry and NMR spectroscopy. Glycoconj. J..

[28] Liu C.H., Li X.D., Li Y.H., Feng Y., Zhou S., Wang F.S. (2008). Structural characterization and antimutagenic activity of a novel polysaccharide isolated from *Sepiella maindroni* ink. Food Chem..

[29] Liu H.Z., Tao Y.X., Luo P., Deng C.M., Gu Y.P., Yang L., Zhong J.P. (2016). Preventive effects of a novel polysaccharide from *Sepia esculenta* ink on ovarian failure and its action mechanisms in cyclophosphamide-treated mice. J. Agric. Food Chem..

[30] Cardoso M.J., Costa R.R., Mano J.F. (2016). Marine origin polysaccharides in drug delivery systems. Mar. Drugs.

[31] De Jesus Raposo M.F., de Morais A.M.B., de Morais R.M.S.C. (2015). Marine polysaccharide from algae with potential biomedical applications. Mar. Drugs.

[32] Vazqez J.A., Rodriguez-Amado I., Montemayor M.I., Fraguas J., del Pilar Gonzalez M., Murado M.A. (2013). Chondronitin sulfate, hyaluronic acid and chitin/chitosan production using marine waste sources: Characteristics, applications and eco-friendly processes: A review. Mar. Drugs.

[33] Takaya Y., Uchisawa H., Matsue H., Okuzaki B.I., Narumi F., Sasaki J.I., Ishida K. (1994). An investigation of the antitumor peptidoglycan fraction from squid ink. Biol. Pharm. Bull..

[34] Tang Q., Zuo T., Lu S., Wu J., Wang J., Zheng R., Chen S., Xue C. (2014). Dietary squid ink polysaccharides ameliorated the intestinal microflora dysfunction in mice undergoing chemotherapy. Food Funct..

[35] Zuo T., Cao L., Sun X., Li X., Wu J., Lu S., Xue C., Tang Q. (2014). Dietary squid ink polysaccharide could enhance SIgA secretion in chemotherapeutic mice. Food Funct..

[36] Lu S., Zuo T., Zhang N., Shi H., Liu F., Wu J., Wang Y., Xue C., Tang Q. (2016). High throughput sequencing analysis reveals amelioration of intestinal dysbiosis by squid ink polysaccharide. J. Funct. Foods.

[37] Zuo T., Cao L., Xue C., Tang Q.J. (2015). Dietary squid ink polysaccharide induces goblet cells to protect small intestinal from chemotherapy induced injury. Food Funct..

[38] Zuo T., He X., Cao L., Xue C., Tang Q.J. (2015). The dietary polysaccharide from *Ommastrephes bartrami* prevents chemotherapeutic mucositis by promoting the gene expression of antimicrobial peptides in Paneth cells. J. Funct. Foods.

[39] Zuo T., Cao L., Li X., Zhang Q., Xue C., Tang Q. (2015). The squid ink polysaccharides protect tight junctions and adherens junctions from chemotherapeutic injury in the small intestinal epithelium of mice. Nutr. Cancer.

[40] Cao L., Zuo T., Li X.M., Yu Q., Cao B.B., Wang J.F. (2013). Protective effects of polysaccharides from the ink of *Ommastrephes bartrami* on the intestinal mucosa of the mice with chemotherapy and its mechanism. Chin. Pharmacol. Bull..

[41] Chen S.G., Wang J.F., Xue C.H., Li H., Sun B.B., Xue Y., Chai W.G. (2010). Sulfation of a squid ink polysaccharide and its inhibitory effect on tumor cell metastasis. Carbohyd. Polym..

[42] Chen S.G., Li Z.G., Wang Y.M., Li G.Y., Sun B.B., Xue C.H. (2010). Sulfation of a squid ink polysaccharide and its anticoagulant activities. Chem. J. Chin. Univ..

[43] Wang S., Cheng Y., Wang F., Sun L., Liu C., Chen G., Li Y., Ward S.G., Qu X. (2008). Inhibition activity of sulfated polysaccharide of *Sepiella maindroni* ink on matrix metalloproteinase (MMP)-2. Biomed. Pharmacother..

[44] Jiang W., Tian W., Ijaz M., Wang F. (2017). Inhibition of EGF-induced migration and invasion by sulfated polysaccharide of *Sepiella maindroni* ink via the suppression of EGFR/Akt/p38 MAPK/MMP-2 signaling pathway in KB cells. Biomed. Pharmacother..

[45] Jiang W., Cheng Y., Zhao N., Li L., Shi Y., Zong A., Wang F. (2018). Sulfated polysaccharide of *Sepiella Maindroni* ink inhibits the migration, invasion and matrix metalloproteinase-2 expression through suppressing EGFR-mediated p38/MAPK and PI3K/Akt/mTOR signaling pathways in SKOV-3 cells. Int. J. Bio. Macromol..

[46] Zong A.Z., Zhao T., Zhang Y., Song X.L., Shi Y.K., Cao H.Z., Liu C.H., Cheng Y.N., Qu X.J., Cao J.C. (2013). Anti-metastatic and anti-angiogenic activities of sulfated polysaccharide of *Sepiella maindroni* ink. Carbohyd. Polym..

[47] Zong A.Z., Liu Y.H., Zhang Y., Song X., Shi Y.K., Cao H.Z., Liu C.H., Cheng Y., Jiang W.J., Du F.L. (2015). Anti-tumor activity and the mechanism of SIP-S: A sulfated polysaccharide with anti-metastatic effect. Carbohyd. Polym..

[48] Luo P., Liu H.Z., Le X.Y., Du H., Kang X.H. (2018). Squid ink polysaccharide prevents chemotherapy induced injury in the testes of reproducing mice. Pak. J. Pharm. Sci..

[49] Le X.Y., Luo P., Gu Y.P., Tao Y.X., Liu H.Z. (2015). Interventional effects of squid ink polysaccharide on cyclophosphamide-associated testicular damage in mice. Bratislava Med. J..

[50] Le X.Y., Luo P., Gu Y.P., Tao Y.X., Liu H.Z. (2015). Squid ink polysaccharide reduces cyclophosphamide-induced testicular damage via Nrf2/ARE activation pathway in mice. Iran. J. Basic Med. Sci..

[51] Gu Y.P., Yang X.M., Duan Z.H., Luo P., Shang J.H., Xiao W., Tao Y.X., Zhang D.Y., Zhang Y.B., Liu H.Z. (2017). Inhibition of chemotherapy-induced apoptosis of testicular cells by squid ink polysaccharide. Exp. Ther. Med..

[52] Gu Y.P., Yang X.M., Duan Z.H., Shang J.H., Luo P., Xiao W., Zhang D.Y., Liu H.Z. (2017). Squid ink polysaccharide prevents autophagy and oxidative stress affected by cyclophosphamide in Leydig cells of mice: A pilot study. Iran. J. Basic Med. Sci..

[53] Gu Y.P., Yang X.M., Luo P., Li Y.Q., Tao Y.X., Duan Z.H., Xiao W., Zhang D.Y., Liu H.Z. (2017). Inhibition of acrolein-induced autophagy and apoptosis by a glycosaminoglycan from *Sepia esculenta* ink in mouse Leydig cells. Carbohyd. Polym..

[54] Liu H.Z., Wang G., Wu J.L., Shi L.S., Zhong J.P., Pan J.Q. (2012). Amelioration of sepia ink polysaccharides on internal organs injured by cyclophosphamide in mice. Chin. J. Mod. Appl. Pharm..

[55] Wu J.L., Wang G., Shi L.S., Zhong J.P., Pan J.Q., Liu H.Z. (2012). Sepia ink polysaccharide preparation attenuates cyclophosphamide toxicity in mice. Chin. J. Mar. Drugs.

[56] Wang G., Zhong J.P., Liu H.Z. (2012). Amelioration of sepia ink polysaccharides on functional inhibition of bone marrow by cyclophosphamide in rats. Sci. Technol. Food Ind..

[57] Cao S.Q., Liu L., Zhu M.F., Chen Q.P., Qi X.Y., Yang H. (2017). Preparation of polysaccharide from sepia ink and its effects on B16F10 cells. J. Nucl. Agric. Sci..

[58] Liu H.Z., Xiao W., Gu Y.P., Tao Y.X., Zhang D.Y., Du H., Shang J.H. (2016). Polysaccharide from *Sepia esculenta* ink and cisplatin inhibit synergistically proliferation and metastasis of triple-negative breast cancer MDA-MB-231 cells. Iran. J. Basic Med. Sci..

[59] Chen W., Zheng R., Baade P.D., Zhang S., Zeng H., Bray F., Jemal A., Yu X.Q., He J. (2016). Cancer statistics in China, 2015. CA Cancer J. Clin..

[60] Senthilkumar S., Yogeeta S.K., Subashini R., Devaki T. (2006). Attenuation of cyclophosphamide induced toxicity by squalene in experimental rats. Chem. Biol. Interact..

[61] Selvakumar E., Prahalathan C., Sudharsan P.T., Varalakshmi P. (2006). Protective effect of lipoic acid on cyclophosphamide-induced testicular toxicity. Clin. Chim. Acta.

[62] Motawi T.M.K., Sadik N.A.H., Refaat A. (2010). Cytoprotective effects of DL-alpha-lipoic acid or squalene on cyclophosphamide-induced oxidative injury: An experimental study on rat myocardium, testicles and urinary bladder. Food Chem. Toxicol..

[63] Aguilar-Mahecha A., Hales B.F., Robaire B. (2005). Effects of acute and chronic cyclophosphamide treatment on meiotic progression and the induction of DNA double-strand breaks in rat spermatocytes. Biol. Reprod..

[64] Elangovan N., Chiou T.J., Tzeng W.F., Chu S.T. (2006). Cyclophosphamide treatment causes impairment of sperm and its fertilizing ability in mice. Toxicology.

[65] Luo P., Liu H.Z. (2013). Antioxidant ability of squid ink polysaccharides as well as their protective effects on DNA damage in vitro. Afr. J. Pharm. Pharmacol..

[66] Yang J., Liu K.X., Qu J.M., Wang X.D. (2013). The changes induced by cyclophosphamide in intestinal barrier and microflora in mice. Eur. J. Pharmacol..

[67] Meng Y., Huang Y.K. (2012). Chemotherapeutics impairment on intestinal mucosal barrier and the prevention and treatment. Med. Recapitul..

[68] Round J.L., Mazmanian S.K. (2009). The gut microbiota shapes intestinal immune responses during health and disease. Nat. Rev. Immunol..

[69] Macpherson A.J., Harris N.L. (2004). Interactions between commensal intestinal bacteria and the immune system. Nat. Rev. Immunol..

[70] Zhang W., Sun Y.L., Chen D.H. (2014). Effects of chitin and sepia ink hybrid hemostatic sponge on the blood parameters of mice. Mar. Drugs.

[71] Huang N., Lin J., Li S., Deng Y., Kong S., Hong P., Yang P., Liao M., Hu Z. (2018). Preparation and evaluation of squid ink polysaccharide-chitosan as a wound-healing sponge. Mat. Sci. Eng. C Mater..

[72] Franz S.A. (1995). Structure-activity relationship of antithrombotic polysaccharide derivatives. Int. J. Bio. Macromol..

